# Theaflavins enhances laying hen production performance and egg antioxidant capacity via improving antioxidant status and regulating lipid metabolism

**DOI:** 10.3389/fvets.2025.1566580

**Published:** 2025-06-18

**Authors:** Ling Zhou, Pinyao Zhao, Jinwei Zhang, Jie Zhong, Yulin Rao, Jia Tang, Longsheng Jiang, Fen Chen, Lijuan Chen, Keying Zhang, Weimin Ouyang, Jialong Peng

**Affiliations:** ^1^Department of Quality Management and Inspection and Quarantine, Yibin University, Yibin, China; ^2^YibinShangougou Agricultural Science and Technology Co., Ltd, Yibin, China; ^3^Institute of Animal Nutrition, Key Laboratory for Animal Disease-Resistance Nutrition of China Ministry of Education, Sichuan Agricultural University, Chengdu, China; ^4^Yibin Agricultural Product Quality Inspection and Testing Center, Yibin, China

**Keywords:** theaflavins, production performance, egg antioxidant capacity, antioxidant status, lipid metabolism, laying hen

## Abstract

**Introduction:**

Theaflavins (TF), natural compounds extracted from black tea, demonstrate various beneficial functions including antioxidant properties and lipid metabolism regulation. However, their effects on laying hens remained unclear. This study investigated the effects of different TF levels on production performance, egg quality, antioxidant capacity, lipid metabolism in laying hens, and egg antioxidant capacity.

**Methods:**

A total of 512 twenty-nine-week-old Lohmann commercial laying hens were randomly divided into four dietary treatments (0, 250, 500, or 1000 mg/kg TF) in a completely randomized design. Each treatment consisted of eight replicates of 16 birds, and the experiment lasted for 8 weeks. Data were analyzed using one-way analysis of variance (ANOVA) followed by Duncan’s multiple range test, with quadratic polynomial contrasts applied to evaluate dose-response relationships.

**Results:**

Results showed that TF supplementation quadratically increased (*p* < 0.05) egg production, egg yolk color, serum total antioxidant capacity (T-AOC), hepatic T-AOC, uterine T-AOC, hepatic superoxide dismutase activity, serum total cholesterol (TC), hepatic TC, hepatic triglycerides, and the expression of antioxidant-related and lipid metabolism-related genes. It also enhanced the 1,1-diphenyl-2-picrylhydrazyl (DPPH) radical scavenging capacity, T-AOC, tyrosine, and tryptophan levels in egg yolks. TF supplementation significantly decreased (*p* < 0.05) malondialdehyde (MDA) levels in serum, liver, ovaries, and egg yolks.

**Conclusion:**

These findings suggest that TF improves laying performance by enhancing antioxidant capacity and regulating lipid metabolism, while simultaneously boosting egg antioxidant potential. Based on the quadratic regression analysis, the optimal TF supplementation level was determined to be 500 mg/kg.

## 1 Introduction

Theaflavins (TF) are key functional phytochemicals formed through the enzymatic action of polyphenol oxidase during the processing of black tea. They constitute 2–20 g/kg of the dry weight of solids in brewed black tea (1). The main structure of TF features a seven-membered benzotropolone ring, followed by decarboxylation and simultaneous fusion with the epicatechin ring B or epicatechin gallate (2). Over 25 types of TF have been identified, with the major characterized structures being theaflavin (TF1), theaflavin 3-gallate (TF2A), theaflavin-3′-allate (TF2B), and theaflavin-3,3′-digallate (TF3).

TF have been widely reported to resist the oxidative stress and regulated the lipid metabolism. Firstly, TF have excellent antioxidant capacity. Yang et al. report that TF exhibit the highest antioxidant activity in the Fenton reaction system, 2,2-diphenyl-1-picrylhydrazyl (DPPH) assay, and H_2_O_2_-mediated oxidative damage in HPF-1 cells, compared to that of other oxidized phenolic compounds in black tea (3). The hydroxyl radical-scavenging ability and DPPH scavenging ability follow the order TF3 > TF2 > TF1 > epigallocatechin-3-gallate (EGCG) (4). Research on diabetic mice reveals that TF could counteract the increase and decrease of malondialdehyde (MDA) and glutathione peroxidase (GSH-*Px*) levels in the liver and kidney tissues, respectively (5). The nuclear factor erythroid 2-related factor 2 (*Nrf2*) pathway is a key regulator of oxidative stress (6). TF upregulate the expression of total *Nrf2*, nuclear *Nrf2*, and its downstream targets heme oxygenase 1 (*HO-1*) and NAD(P)H quinone dehydrogenase 1 (*NQO1*), protecting the liver and kidneys of mice from oxidative damage (4). Second, TF regulate lipid metabolism, improving hyperlipidemia and regulating body composition in human studies. TF decrease the serum levels of total cholesterol (TC), triglyceride (TG), and low-density lipoprotein cholesterol in obese rats by 26.5, 50.8, and 71.7%, respectively (7). This effect is mainly attributed to the ability of TF to inhibit lipid synthesis and catabolism. TF decrease the mRNA and protein expression levels of fatty acid synthase (*FAS*), acetyl-CoA carboxylase (*ACC*), and HMG-CoA reductase while increasing the expression levels of carnitine palmitoyl transferase 1 (*CPT1*), thereby inhibiting fatty acid and cholesterol synthesis and decreasing lipid accumulation (8, 9). In our previous study, dietary supplementation with 600 mg/kg tea polyphenols was observed to alleviate oxidative stress induced by aging corn in laying hens. However, the potential for TF to demonstrate superior antioxidant efficacy in this context remains unclear, warranting further comparative investigation (10).

Currently, the research on the physiological functions of TF is already very extensive, and it has been demonstrated that they have the effects of enhancing antioxidant capacity and regulating lipid metabolism. However, it is unclear whether TF can be applied in laying hens, what their effects are on the production performance of laying hens and the antioxidant capacity of eggs, and what the optimal level of addition is for laying hens. Thereby, this study was conducted to investigate the effects of different levels of TF on production performance, egg quality, antioxidant capacity, lipid metabolism in laying hens, and the antioxidant capacity of eggs. We hypothesized that 500 mg/kg TF would increase the performance, egg quality, antioxidant status and egg antioxidant capacity of laying hens.

## 2 Materials and methods

### 2.1 Theaflavins

TF were purchased from Dannisi Technology Co., Ltd. (Shan’xi province, China), with 62.9% purity. The composition includes 40.67% TF1, 8.91% TF3, 7.14% TF2A, and 6.18% TF2B.

### 2.2 Animals and diets

This study was performed on a poultry farm in Yi’bin, China, during the summer (Latitude: 28.7599°N, Longitude: 104.6399°E). All experimental procedures were approved by the Animal Care and Use Committee of Yibin University (No. 202402). Twenty-nine-week-old (*n* = 512) Lohmann commercial laying hens were randomly allocated to one of the four dietary treatments. Each treatment included eight replicates, with 16 hens per replicate, housed in two cages containing eight birds each. The housing temperature and relative humidity were 20 ± 4°C and 50–65%, respectively. The four treatment groups consisted of a control group (basal diet) and three experimental groups with different levels of TF addition (basal diet + 250, 500, 1,000 mg/kg TF). The diets (Table 1) were based on corn and soybean meal, with nutrient levels aligned with the Agricultural Trade Standardization of China (11). Feed and water were supplied ad libitum, and a 16L:8D photoperiod was used during the 8-week laying hens trial.

**Table 1:** Composition and nutrient levels of the experimental diets (fed-basis).

Ingredient	g/kg	Nutrient content	g/kg^2^
Corn	582.1	Metabolizable energy, kcal/kg	2,689.76
Soybean meal	245.0	Crude protein	165.1
Soybean oil	20.0	Calcium	37.1
Distillers dried grains with solubles	17.4	Available phosphorus	3.7
Cottonseed meal	20.0	Lysine	7.4
Sodium chloride	3.0	Methionine	3.4
Calcium carbonate	87.0	Threonine	5.5
Calcium hydrophosphate	14.5	Tryptophan	1.7
Premix^1^	10.0		
DL-Methionine	1.0		

1 The premix provided the following (per kilogram of diet): vitamin A, 9,750 IU; vitamin D3, 2,200 IU, vitamin E, 5.0 IU; vitamin B1, 2.5 mg; vitamin B2, 6.25 mg; vitamin B6, 3.75 mg; vitamin B12, 0.019 mg; D-pantothenic acid, 12.25 mg; nicotinic acid, 40 mg; biotin, 0.3 mg; iron, 80 mg; copper, 10 mg; manganese, 120 mg; zinc, 80 mg; selenium, 0.3 mg; iodine, 1.0 mg; choline chloride, 450 mg.

2 Calculated value.

### 2.3 Hen performance

Daily records were kept of egg production, egg weight, and the quantity of dirty and broken eggs. The laying rate, average egg weight, and rates of dirty and broken eggs were then computed. Weekly feed intake (ADFI) was monitored, and the feed conversion rate (FCR) was determined.

### 2.4 Egg collection

At the end of the trial, a total of 128 eggs for each treatment, with four eggs in each replicate, were gathered and split into two groups. One half of the eggs was utilized for assessing egg quality. While the other half were used for measuring the antioxidant capacity of the egg yolks (two pooled egg yolks per replicate).

### 2.5 Sample collection

In the final days of the trial, 10 mL blood samples (*n* = 8) were taken from the brachial vein of one laying hen per replicate and placed in lithium heparinized vacutainers. The serum was separated at 4°C and then stored at −20°C for analysis of antioxidant status. After the blood collection, the chicks were euthanized by cervical dislocation, and the liver and ovaries were promptly extracted, frozen at −80°C, and stored for antioxidant status analysis.

### 2.6 Egg quality

The Multi-tester (EMT-7300, Robotmation Co., Ltd., Tokyo, Japan) was utilized to measure the albumen height, Haugh unit, and color of the egg yolk. The yolk and eggshell (which had been dried at room temperature for 3 days) were meticulously separated and weighed. The ratios of the eggshell, albumen, and yolk within the egg were then calculated. The thickness of the eggshell (excluding the inner and outer membranes) was measured using Vernier calipers, with the average taken from three measurements at different locations (top, middle, and bottom).

### 2.7 Antioxidant status of laying hens

**Antioxidant enzyme activity.** Liver and ovarian tissue samples (*n* = 8) were homogenized in PBS and centrifuged at 1,699×*g* for 10 min. The supernatant was then collected for the determination of correlation indices. The activities of SOD, total antioxidant capacity (T-AOC), malondialdehyde (MDA) levels in the liver, ovarian tissue, and serum, as well as TC and TG in the liver and serum, were analyzed using reagent kits from Jiancheng Bioengineering Institute in Jiangsu, China. SOD activity was assessed using the hydroxylamine method, where superoxide anions generated from the xanthine–xanthine oxidase system oxidized hydroxylamine to produce nitrite, which was measured at 550 nm using a colorimetric approach. The production of nitrite was inhibited by SOD, indicating the activity of superoxide anions. One unit of SOD is defined as the amount of sample that inhibits 50% of the activity under the assay conditions, and SOD activity was reported as units per gram of tissue. T-AOC was evaluated using the ferric reducing-antioxidant power assay, conducted within a 5-min timeframe at 37°C and measured at 593 nm. MDA levels were determined using the thiobarbituric acid method, where 1 mL of the homogenate was treated with thiobarbituric acid, mixed quickly, and heated in a boiling water bath for 40 min. The samples were then centrifuged at 10,621×*g* for 10 min, and the supernatants were placed in a 96-well plate and measured at 532 nm.

**Antioxidant-related gene expression.** Total RNA was extracted from liver tissue using Trizol reagent (Takara, Dalian, China), according to the protocol of the manufacturer. The absorbance ratio at 260–280 nm was calculated for each sample. cDNA synthesis was conducted using the primeScript RT reagent kit (Takara), and real-time PCR was performed using the SYBR Premix Ex Tap (Takara). The PCRs were conducted on an Applied Biosystems 7900HT Real-Time PCR system (Applied Biosystems, Foster City, CA, USA). The amplification cycles included an initial denaturation at 95°C for 35 s, followed by 40 cycles of denaturation at 95°C for 5 s, annealing at 60°C for 34 s, and extension at 95°C for 15 s, and a final elongation step at 60°C for 15 s. Table 2 presents the primer sequences for antioxidant-related genes.

**Table 2:** Gene-specific primers for real-time quantitative reverse transcription PCR.

Genes	Primers (5′–3′)
*Nrf2*	Forward: TGTGTGTGATTCAACCCGACT
	Reverse: TTAATGGAAGCCGCACCACT
*NQO1*	Forward: GTTCAATGCCGTGCTCTCAC
	Reverse: CCGCTTCAATCTTCTTCTGC
*HO-1*	Forward: TTGGCAAGAAGCATCCAGA
	Reverse: TCCATCTCAAGGGCATTCA
*FAS*	Forward: ACTGTGGGCTCCAAATCTTCA
	Reverse: CAAGGAGCCATCGTGTAAAGC
*ACC*	Forward: TGTGGCTGATGTGAGCTTTC
	Reverse: ACTGTCGGGTCACCTTCAAC
*CPT1*	Forward: GAGAAGAGTGCAGTGAGAAGAG
	Reverse: CCAGCCACAGAAGTAGAGTAAG

Nrf2 = nuclear factor erythroid 2-related 2; NQO1 = NAD(P)H: quinone dehydrogenase 1; HO-1 = heme oxygenase-1; FAS = fatty acid synthase; ACC = dacetyl-CoA carboxylase; CPT1 = carnitine palmitoyl transferase 1.

### 2.8 Lipid metabolism index of laying hens

**TG and TC.** TC and TG (*n* = 8) in liver and serum were analyzed using reagent kits (Jiancheng Bioengineering Institute, Jiangsu, China), and the supernatant was used for antioxidant status analysis. TG and TC were determined using the glycerol-3-phosphate oxidase peroxidase method and measured at 500 nm.

**Lipid metabolism-related gene expression.** The liver FAS, ACC, HMGCR, and CPT1 were measured according to the methods described for antioxidant-related gene expression analysis. Table 2 shows the primer sequences for lipid metabolism-related genes.

### 2.9 Antioxidant capacity of eggs

**Free radical-scavenging activity on 1,1-diphenyl-2-picrylhydrazyl (DPPH).** The scavenging effect of the DPPH free radical (*n* = 8) was measured using a previously described method (12). A sample solution (1 mL, containing 10 mg of egg yolk per mL; with ethanol as a control) was combined with an equal volume of a DPPH (0.1 mM) solution, mixed thoroughly, and left to incubate for 30 min at room temperature. The absorbance was measured at 517 nm. The scavenging effect was calculated using this formula: DPPH scavenging activity (%) = [(Blank absorbance − Sample absorbance) /Blank absorbance] × 100.

**MDA.** The egg yolk samples (*n* = 8) were homogenized in PBS and centrifuged at 1,699×*g* for 10 min. The resulting supernatant was collected for MDA determination. MDA content in egg yolk was measured as described in the tissue antioxidant status analysis.

**SOD and T-AOC.** SOD activity and T-AOC (*n* = 8) in egg yolks were measured as described in the tissue analysis methods.

### 2.10 Egg composition

**Free amino acid.** Free amino acids were determined using the method described by Nimalaratne et al. (13) with slight modifications (*n* = 8). About 100 mg of freeze-dried egg yolk was mixed with 1 mL of a 10% sulfosalicylic acid solution, vortexed, and then centrifuged at 15,294×*g* for 10 min using a Centrifuge 5430/R from Eppendorf in Hamburg, Germany. The resulting supernatant was passed through a 0.22-μm nylon syringe filter prior to analysis with an automated AA analyzer (L-8900, Hitachi, Tokyo, Japan) equipped with a lithium high-performance column.

**Fatty acid profile.** The analysis of the egg yolk (*n* = 8) was measured according to the national standard method (14). Fatty acid methyl esters were analyzed using a gas chromatograph (7890A, Agilent Technologies, Santa Clara, CA, USA) that featured a flame ionization detector and a silica capillary column (Supelco SP-2560; 100 × 0.25 mm i.d., 0.20 μm film thickness). Nitrogen was used as the carrier gas at a pressure of 0.5 kg/cm^2^. The injector and detector temperatures were kept at 250°C. The findings were reported as percentages (%) of the total fatty acids.

### 2.11 Statistical analysis

The results were statistically analyzed using one-way ANOVA followed by Duncan’s multiple range tests of SPSS 27.0 (SPSS Inc., Chicago, IL, USA). Regression analysis was subsequently performed to evaluate linear and quadratic effects. Differences among treatments were considered significant at *p* < 0.05, and results were reported as mean and standard error mean (SEM).

## 3 Results

### 3.1 Hen performance

Dietary TF levels had no significant effect on hen performance, including egg weight, ADFI, FCR, as well as dirty and broken egg rate (Table 3). Increasing TF levels quadratically (*p* < 0.05) increased egg production throughout the entire laying period.

**Table 3:** Production performance of laying hens at different theaflavin levels (*n* = 8)^1,2^.

Items	CON	TF250	TF500	TF1000	SEM	*p*-value	Linear	Quadratic
Egg production (%)	95.48^b^	95.76^ab^	97.68^a^	94.44^b^	0.40	<0.05	0.377	<0.05
Egg weight (g)	60.67	60.77	61.29	61.45	0.25	0.632	0.213	0.454
ADFI (g)	117.21	117.68	120.95	117.98	1.42	0.801	0.787	0.715
FCR	2.02	2.02	2.02	2.03	0.02	0.997	0.843	0.978
Dirty egg (%)	6.95	7.46	7.50	7.24	0.39	0.961	0.859	0.867
Broken egg (%)	0.90	0.77	0.66	0.70	0.07	0.711	0.350	0.500

^a,b^Different superscripts in a row indicate a significance difference (*p* < 0.05).

^1^Each value represents the mean value of 8 replicates per treatment.

^2^CON = control group; TF250 = CON + 250 mg/kg theaflavins; TF500 = CON + 500 mg/kg theaflavins; TF1000 = CON + 1,000 mg/kg theaflavins; ADFI = average daily feed intake; FCR = feed conversion rate; SEM = standard error of the mean.

### 3.2 Egg quality

Dietary TF levels had no significant effect on egg quality, including eggshell color, egg strength, eggshell thickness, albumen height, and Haugh units (Table 4). However, increasing TF levels quadratically (*p* < 0.05) increased the egg yolk color.

**Table 4:** Production performance of laying hens at different theaflavin levels (*n* = 8)^1,2^.

Items		CON	TF250	TF500	TF1000	SEM	*p*-value	Linear	Quadratic
Eggshell strength (N)		49.25	46.23	49.75	47.19	0.86	0.422	0.661	0.904
Eggshell thickness (mm)		0.35	0.35	0.36	0.35	0.00	0.764	0.758	0.953
Albumen height		7.90	7.91	7.88	8.14	0.10	0.808	0.401	0.624
Haugh unit		87.17	87.65	87.50	89.59	0.59	0.468	0.139	0.295
Yolk color		9.31^b^	10.19^ab^	10.56^a^	9.44^b^	0.13	<0.05	0.564	<0.05
Albumen height (%)		62.24	62.90	62.88	62.06	0.19	0.268	0.511	0.144
Yolk weight (%)		27.46	27.22	27.42	27.35	0.18	0.186	0.591	0.092
Eggshell weight (%)		10.17	9.97	10.09	10.28	0.07	0.950	0.667	0.845
Eggshell color	L*	77.95	77.23	76.66	77.00	0.41	0.733	0.433	0.527
	a*	7.07	7.84	7.30	7.25	0.25	0.728	0.934	0.809
	b*	15.27	16.48	15.51	14.99	0.34	0.452	0.468	0.488

^a, b^Different superscripts in a row indicate a significance difference (*p* < 0.05).

^1^Each value represents the mean value of 16 replicates per treatment.

^2^CON = control group; TF250 = CON + 250 mg/kg theaflavins; TF500 = CON + 500 mg/kg theaflavins; TF1000 = CON + 1,000 mg/kg theaflavins; L* = lightness; a* = redness; b* = yellowness. SEM = standard error of the mean.

### 3.3 Antioxidant status of laying hens

**Antioxidant enzyme activity assay.** TF significantly enhanced the antioxidant capacity of laying hens (Table 5). Increasing TF levels quadratically (*p* < 0.05) increased the serum T-AOC, liver T-AOC, uterus T-AOC, and liver SOD, while it quadratically (*p* < 0.05) decreased the MDA levels in the serum, liver, and ovary of laying hens.

**Table 5:** Antioxidant status of laying hens at different theaflavin levels (*n* = 8)^1,2^.

Items	CON	TF250	TF500	TF1000	SEM	*p*-value	Linear	Quadratic
Serum								
T-AOC	0.79^b^	1.18^ab^	1.39^a^	1.07^ab^	0.07	<0.05	0.24	<0.05
SOD	51.81	52.35	53.16	50.73	1.17	0.914	0.73	0.78
MDA	1.91^b^	1.65^b^	1.27^a^	1.63^b^	0.07	<0.05	0.13	<0.05
Liver								
T-AOC	1.45^b^	1.82^ab^	2.64^a^	1.63^b^	0.17	<0.05	0.68	<0.05
SOD	238.56^b^	261.60^ab^	305.98^a^	237.73^b^	9.54	<0.05	0.97	<0.05
MDA	12.96^a^	12.87^a^	10.02^b^	11.59^ab^	0.41	<0.05	0.12	0.07
Ovary								
T-AOC	1.38	1.49	1.54	1.36	0.05	0.535	0.77	0.33
SOD	77.72	73.42	72.07	70.02	2.47	0.748	0.30	0.54
MDA	1.11^a^	0.83^ab^	0.60^b^	1.08^a^	0.07	<0.05	0.99	<0.05
Uterus								
T-AOC	3.11^b^	3.88^ab^	4.75^a^	2.79^b^	0.27	<0.05	0.59	<0.05
SOD	142.45	161.77	153.64	140.96	6.00	0.590	0.68	0.48
MDA	37.70	35.66	30.60	32.30	1.21	0.151	0.08	0.10

^a, b^Different superscripts in a row indicate a significance difference (*p* < 0.05).

^1^Each value represents the mean value of 8 replicates per treatment.

^2^CON = control group; TF250 = CON + 250 mg/kg theaflavins; TF500 = CON + 500 mg/kg theaflavins; TF1000 = CON + 1,000 mg/kg theaflavins; T-AOC = total antioxidant capacity; SOD = superoxide dismutase; MDA = malondialdehyde; SEM = standard error of the mean.

**Figure 1:**
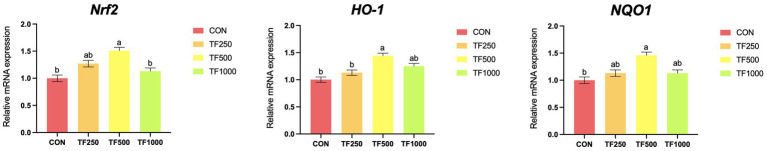
Antioxidant-related gene expression of laying hens at different theaflavin levels. ^a,b^Different superscripts indicate a significance difference (*p* < 0.05). Nrf2 = nuclear factor erythroid 2-related 2; NQO1 = NAD(P)H: quinone dehydrogenase 1; HO-1 = heme oxygenase-1. ^1^Values are means ± SEM. Each value represents the mean value of 8 replicates per treatment. ^2^All the data were acquired using real-time PCR.

**Antioxidant-related gene expression.** Figure 1 illustrates the expression of antioxidant-related genes. Increasing TF levels quadratically (*p* < 0.05) increased the expression of *Nrf2*, *HO-1*, and *NQO1* in the liver of laying hens.

### 3.4 Lipid metabolism indexes of laying hen

**TG and TC.** Table 6 shows the lipid metabolism indices in serum and liver. Increasing TF levels quadratically (*p* < 0.05) decreased the serum TC, as well as the liver TC and TG content in laying hens.

**Table 6:** Lipid metabolism indexes of laying hens at different theaflavin levels (*n* = 8)^1,2^.

Items	CON	TF250	TF500	TF1000	SEM	*p*-value	Linear	Quadratic
Serum								
TG	14.99	14.01	14.10	14.57	0.34	0.738	0.833	0.573
TC	4.57^a^	3.78^ab^	2.95^b^	3.65^ab^	0.20	<0.05	0.104	<0.05
Liver								
TG	1.14^a^	1.00^ab^	0.62^b^	1.12^a^	0.08	<0.05	0.887	<0.05
TC	1.12^a^	0.64^b^	0.60^b^	0.69^b^	0.08	0.041	0.094	<0.05

^a,b^ Different superscripts in a row indicate a significance difference (*p* < 0.05).

^1^Each value represents the mean value of 8 replicates per treatment.

^2^CON = control group; TF250 = CON + 250 mg/kg theaflavins; TF500 = CON + 500 mg/kg theaflavins; TF1000 = CON + 1,000 mg/kg theaflavins; TG = triglyceride; TC = total cholesterol; SEM = standard error of the mean.

**Figure 2:**
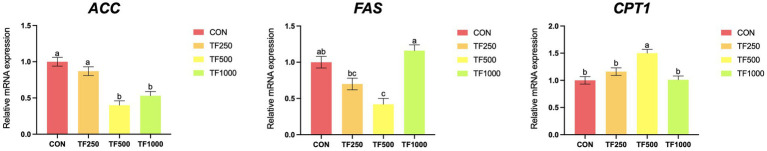
Lipid metabolism-related gene expression of laying hens at different theaflavin levels. ^a,b^Different superscripts indicate a significance difference (*p* < 0.05). ACC = acetyl-CoA carboxylase; FAS = fatty acid synthase; CPT1 = carnitine palmitoyl transferase 1. ^1^Values are means ± SEM. Each value represents the mean value of 8 replicates per treatment. ^2^All the data were acquired using real-time PCR.

**Lipid metabolism-related gene expression.** Figure 2 depicts the expression of lipid metabolism-related genes. Increasing TF levels quadratically (*p* < 0.05) decreased the expression of *FAS* and *ACC* while increasing the expression of *CPT1* (*p* < 0.05) in the liver of laying hens.

### 3.5 Antioxidant capacity of eggs

Table 7 shows the antioxidant capacity of eggs. Increasing TF levels quadratically (*p* < 0.05) increased the DPPH value of egg yolks. TF (500 mg/kg) significantly (*p* < 0.05) decreased the egg yolk MDA content compared to those of the other groups and significantly (*p* < 0.05) increased the egg yolk T-AOC compared to that of the control group.

**Table 7:** Antioxidant capacity of egg yolks at different theaflavin levels (*n* = 8)^1,2^.

Items	CON	TF250	TF500	TF1000	SEM	*p*-value	Linear	Quadratic
MDA	118.88^a^	106.96^a^	89.45^b^	106.54^a^	4.57	0.150	0.341	0.089
DPPH	22.74^b^	23.06^b^	25.14^a^	23.21^b^	0.29	<0.05	0.409	<0.05
T-AOC	3.86^b^	4.08^ab^	5.36^a^	4.17^ab^	0.24	0.097	0.522	0.121
SOD	272.92	266.74	282.69	284.92	14.25	0.970	0.693	0.926

^a,b^Different superscripts in a row indicate a significance difference (*p* < 0.05).

^1^Each value represents the mean value of 8 replicates per treatment.

^2^CON = control group; TF250 = CON + 250 mg/kg theaflavins; TF500 = CON + 500 mg/kg theaflavins; TF1000 = CON + 1,000 mg/kg theaflavins; MDA = malondialdehyde; DPPH = free radical-scavenging activity on 1,1-diphenyl-2-picrylhydrazyl; T-AOC = total antioxidant capacity; SOD = superoxide dismutase; SEM = standard error of the mean.

### 3.6 Egg composition

**Free amino acids.** Table 8 shows the free amino acids in egg yolks. Increasing TF levels quadratically (*p* < 0.05) increased the tyrosine and tryptophan content in egg yolks.

**Table 8:** Free amino acids of egg yolks at different theaflavin levels (*n* = 8)^1,2^.

Items	CON	TF250	TF500	TF1000	SEM	*p*-value	Linear	Quadratic
Aspartic	246.75	265.44	214.07	234.08	10.37	0.355	0.414	0.636
Threonine	366.51	358.15	354.93	358.61	7.32	0.961	0.764	0.861
Serine	410.66	400.95	412.05	429.14	7.28	0.593	0.247	0.420
Glutamic	1,087.14	1,084.46	1,098.56	1,107.33	14.67	0.947	0.569	0.851
Glycine	127.43	120.87	123.14	131.67	3.78	0.765	0.532	0.580
Alanine	144.31	148.84	158.99	149.62	3.52	0.539	0.597	0.407
Valine	321.69	327.92	327.64	314.21	3.89	0.569	0.368	0.359
Methionine	173.01	173.74	178.11	171.49	1.97	0.681	0.798	0.569
Isoleucine	278.09	282.85	282.55	266.49	3.13	0.203	0.112	0.096
Leucine	649.48	663.53	662.29	634.58	6.12	0.297	0.239	0.155
Tyrosine	419.16^b^	436.61^b^	468.37^a^	431.25^b^	4.74	<0.05	0.424	<0.05
Phenylalanine	474.72	511.75	492.03	466.79	7.99	0.191	0.342	0.173
Histidine	105.01	101.60	108.48	90.28	3.31	0.230	0.127	0.174
Lysine	578.10	634.91	648.15	628.48	12.11	0.214	0.263	0.109
Arginine	441.87	474.10	522.12	503.98	13.66	0.188	0.107	0.104
Tryptophan	155.96^c^	178.34^ab^	184.40^a^	160.99^bc^	3.60	<0.05	0.943	<0.05

^a,b,c^ Different superscripts in a row indicate a significance difference (*p* < 0.05).

^1^Each value represents the mean value of 8 replicates per treatment.

^2^CON = control group; TF250 = CON + 250 mg/kg theaflavins; TF500 = CON + 500 mg/kg theaflavins; TF1000 = CON + 1,000 mg/kg theaflavins; SEM = standard error of the mean.

**Fatty acid profile.** Table 9 shows the fatty acid profile in egg yolks. TF (250 mg/kg) significantly (*p* < 0.05) increased the linoleic acid content compared to that of the 500 and 1,000 mg/kg TF group and increased the eicosenoic acid content compared to that of the 1,000 mg/kg TF group of egg yolks. Additionally, TF linearly (*p* < 0.05) decreased the arachidic acid content in egg yolks.

**Table 9:** Fatty acid profiles of egg yolks at different theaflavin levels (*n* = 8)^1,2^.

Items	CON	TF250	TF500	TF1000	SEM	*p*-value	Linear	Quadratic
Myristic acid (C14:0)	0.36	0.36	0.36	0.36	0.00	0.949	0.596	0.850
Palmitic acid (C16:0)	28.46	28.84	28.16	27.97	0.18	0.343	0.170	0.371
Stearic acid (C18:0)	10.45	10.83	10.66	10.89	0.10	0.416	0.206	0.418
Oleic acid (C18:1)	38.60	39.35	39.34	39.23	0.36	0.875	0.635	0.733
Linoleic acid (C18:2)	17.64^ab^	18.51^a^	17.14^b^	17.07^b^	0.23	0.104	0.141	0.318
Arachidic acid (C20:0)	0.14^a^	0.13^ab^	0.11^b^	0.10^b^	0.01	0.051	<0.05	<0.05
Eicosenoic acid (C20:1)	0.57^ab^	0.61^a^	0.55^ab^	0.48b	0.02	0.153	0.054	0.101
Erucic acid (C22:1)	2.51	2.62	2.49	2.61	0.03	0.165	0.407	0.687
Nervonic acid (C24:1)	0.26	0.21	0.20	0.27	0.02	0.571	0.704	0.360
Docosahexaenoic acid (C22:6)	1.01	1.05	1.00	1.03	0.01	0.581	0.802	0.931
Saturated fatty acid	39.41	40.17	39.29	39.31	0.23	0.498	0.554	0.767
Unsaturated fatty acid	60.59	62.35	60.71	60.69	0.43	0.429	0.684	0.729

^a,b,c^ Different superscripts in a row indicate a significance difference (*p* < 0.05).

^1^Each value represents the mean value of 8 replicates per treatment.

^2^CON = control group; TF250 = CON + 250 mg/kg theaflavins; TF500 = CON + 500 mg/kg theaflavins; TF1000 = CON + 1,000 mg/kg theaflavins; SEM = standard error of the mean.

## 4 Discussion

TF, present in black, red, and oolong teas, exhibit various biological activities and health benefits (15). In this study, we observed that 500 mg/kg TF significantly enhanced the production performance of laying hens. Although reports on the application of TF in laying hens are unknown, other tea extracts, such as theabrownins (TB) and EGCG, have been widely investigated. Studies show that 200–400 mg/kg TB and EGCG enhance the production performance of laying hens (16–18). Furthermore, tea polyphenols (600 mg/kg) could counteract the adverse effects of aging corn and heavy metals on the production performance and egg albumen quality of laying hens (10, 19, 20). Both TF and TBs are key pigments in black tea, derived from the oxidation of catechins and their gallates during the fermentation process. Structurally, TF are dimeric polyphenols with a benzotropolone core (C_17_H_16_O_6_), enabling stronger electron delocalization and antioxidant capacity than the polymeric TBs (irregular catechol-quinone frameworks) or monomeric EGCG (C_22_H_18_O_11_) (21). Therefore, TF exhibit higher antioxidant activity than TBs and hold greater potential for application in the egg production industry (4).

Egg yolk color plays a significant role in the purchasing decisions of consumers. The typical yellow color results from the accumulation of lutein and carotenoids in the diet (22). Tea leaves contain chlorophyll, lutein, carotenoids, and other pigments (22). The TF obtained in this experiment may contain residual lutein and carotenoids that were present during the extraction process of TF, which could increase their deposition in eggs. In China, consumers prefer eggs with a more intense yolk color. The increased yolk color indicates that TF-fed layers may be more preferred.

TF is the main antioxidant active components of black tea and have been shown to effectively improve the antioxidant status in different cell cultures and animal models (23). Our results showed that TF quadratically increased the activities of antioxidant enzymes and decreased the MDA content in laying hens. Ai et al. report that TF could increase the activities of SOD and decrease the MDA levels in serum, thereby alleviating oxidative stress caused by ovariectomy in mice (24). Zeng et al. report that TF administration significantly decreases MDA levels, increases SOD activity, and alleviates oxidative damage in the aorta caused by high-fat diet exposure in mice. Our findings align with that of a previous study demonstrating that TF intake enhances antioxidant capacity in laying hens (5).

*Nrf2* is a key transcription factor that regulates cellular antioxidant defenses against oxidative stress by controlling the expression of various antioxidant and detoxifying enzymes. *NQO1* and *HO-1*, downstream genes of *Nrf2*, are widely recognized for their strong antioxidant properties. Reactive oxygen species (ROS) are highly unstable oxygen-free radicals. TF could suppress receptor activators of nuclear factor-kappa B ligand-induced ROS generation by upregulating the expression of antioxidant genes, such as *Nrf2*, *HO-1*, and catalase in osteoclasts (5, 25, 26). In this study, TF levels quadratically (*p* < 0.05) increased the expression of *Nrf2*, *HO-1*, and *NQO1*. These findings indicate that TF may exert antioxidant effects by upregulating the expression of *HO-1* and *NQO1* genes, with the Nrf2 signaling pathway likely serving as an underlying mechanism.

The role of TF in preventing diabetes has been extensively investigated. TF could regulate energy metabolism, enhance fatty acid oxidation, and reduce *de novo* lipogenesis through the AMP-activated protein kinase (AMPK) signaling pathway (27). In this study, TF quadratically decreased the serum TC, liver TC, and TG content. In obese mice, TF reduce the adiposity index by 24.5% and significantly decrease serum levels of TC and TG by 26.5 and 50.8%, respectively (28). Clinical trials show that oral administration of TF reduces serum levels of TC, TG, and LDL in patients with hypercholesterolemia. TF also improve body composition by proportionally decreasing total and subcutaneous fat while increasing skeletal muscle (29–31). Our finding was consistent with that of a previous study showing that the intake of TF by laying hens regulates energy metabolism and reduces TG and TC content in the serum and liver. By reducing lipid deposition, TF may redirect metabolic energy toward egg synthesis rather than adipose storage, thereby improving feed efficiency and egg production (32). Additionally, enhanced fatty acid oxidation via AMPK activation could maintain hepatic health, ensuring sufficient yolk precursor (VLDL) synthesis for egg formation (33). This dual modulation of energy partitioning and reproductive resource allocation may explain the improved laying performance observed in TF-supplemented hens.

AMPK serves as a key regulator of lipid metabolism, controlling whole-body metabolism by downregulating the *ACC* and *CPT-1* pathways, which regulate beta-oxidation (34). Our results showed that TF quadratically decreased the expression of *FAS* and *ACC* while increasing *CPT1* expression. TF reduced the gene expression of *ACC* and *FAS* while increasing *CPT1* expression in mice liver. This regulation inhibits fatty acids and cholesterol synthesis, decreases lipid accumulation, and promotes fatty acid oxidation (8, 9, 35). Our findings align with this, indicating that TF regulate lipid metabolism in laying hens through the modulation of *ACC*, *FAS*, and *CPT1*, with AMPK serving as a potential signaling pathway.

The antioxidant activity of eggs was determined using the DPPH, T-AOC and MDA contents. The DPPH radical scavenging model and T-AOC are widely used to evaluate the free radical scavenging ability of natural compounds (36). In our study, TF (500 mg/kg) increased the DPPH value and T-AOC while decreasing the MDA content in egg yolks. Studies show that tea extracts increase the antioxidant capacity of eggs. Zhou et al. and Wang et al. report that 600 mg/kg tea polyphenols increased the DPPH value and most free amino acids (isoleucine, leucine, tyrosine, phenylalanine, tryptophan, lysine, threonine, serine, glutamic acid, valine, and methionine) in egg yolks (10, 16). Wang et al. report that 400 mg/kg TBs improved the DPPH value, reducing power value, T-AOC, and levels of tryptophan, cysteine, methionine, and histidine in egg yolks while decreasing the MDA content (17). Our findings are consistent with those of previous studies, which show that antioxidative compounds such as egg-derived peptides, tryptophan, tyrosine, vitamin E, and carotenoids contribute to the T-AOC of egg yolks (13). Our results showed that 500 mg/kg of TF increased the tyrosine and tryptophan content in egg yolks. This indicates that TF enhance the antioxidant capacity of eggs by increasing the content of tyrosine and tryptophan in egg yolks. However, the mechanism by which TF increase the content of tyrosine and tryptophan in egg yolks remains unclear and requires further research. Based on our findings, TF (250 mg/kg) significantly increased the linoleic acid content compared to that of the 500 and 1,000 mg/kg TF group. Studies show that high antioxidant capacity in the yolk preserves lipid quality by preventing oxidation and inhibiting the degradation of certain unsaturated fatty acids (37, 38). This indicates that TF can inhibit the degradation of unsaturated fatty acids by enhancing the antioxidant capacity of eggs.

## 5 Conclusion

TF improved the laying rate of laying hens by enhancing their antioxidant capacity and regulating lipid metabolism. TF also enhanced the antioxidant capacity of eggs by increasing the content of tyrosine and tryptophan levels in egg yolks. The optimal supplementation level of TF in laying hens is 500 mg/kg.

## Data Availability

The datasets presented in this study can be found in online repositories. The names of the repository/repositories and accession number(s) can be found in the article/supplementary material.
